# Viral delivery of *C9orf72* hexanucleotide repeat expansions in mice leads to repeat-length-dependent neuropathology and behavioural deficits

**DOI:** 10.1242/dmm.029892

**Published:** 2017-07-01

**Authors:** Saul Herranz-Martin, Jayanth Chandran, Katherine Lewis, Padraig Mulcahy, Adrian Higginbottom, Callum Walker, Isabel Martinez-Pena y Valenzuela, Ross A. Jones, Ian Coldicott, Tommaso Iannitti, Mohammed Akaaboune, Sherif F. El-Khamisy, Thomas H. Gillingwater, Pamela J. Shaw, Mimoun Azzouz

**Affiliations:** 1 Department of Neuroscience, Sheffield Institute for Translational Neuroscience (SITraN), The University of Sheffield, 385A Glossop Road, Sheffield S10 2HQ, UK; 2 Department of Molecular Biology and Biotechnology, Krebs and Sheffield Institute for Nucleic Acids, Firth Court, University of Sheffield, Sheffield S10 2TN, UK; 3 Molecular, Cellular and Developmental Biology, University of Michigan, 830 North University, Ann Arbor, MI 48109-1048, USA; 4 Centre for Integrative Physiology & Euan MacDonald Centre for Motor Neurone Disease Research, Hugh Robson Building, The University of Edinburgh, 15 George Square, Edinburgh EH8 9XD, UK

**Keywords:** *C9orf72*, Neurodegeneration, ALS/FTD, Mouse model, C9RAN aggregates

## Abstract

Intronic GGGGCC repeat expansions in *C9orf72* are the most common genetic cause of amyotrophic lateral sclerosis (ALS) and frontotemporal dementia (FTD). Two major pathologies stemming from the hexanucleotide RNA expansions (HREs) have been identified in postmortem tissue: intracellular RNA foci and repeat-associated non-ATG dependent (RAN) dipeptides, although it is unclear how these and other hallmarks of disease contribute to the pathophysiology of neuronal injury. Here, we describe two novel lines of mice that overexpress either 10 pure or 102 interrupted GGGGCC repeats mediated by adeno-associated virus (AAV) and recapitulate the relevant human pathology and disease-related behavioural phenotypes. Similar levels of intracellular RNA foci developed in both lines of mice, but only mice expressing 102 repeats generated *C9orf72* RAN pathology, neuromuscular junction (NMJ) abnormalities, dispersal of the hippocampal CA1, enhanced apoptosis, and deficits in gait and cognition. Neither line of mice, however, showed extensive TAR DNA-binding protein 43 (TDP-43) pathology or neurodegeneration. Our data suggest that RNA foci pathology is not a good predictor of *C9orf72* RAN dipeptide formation, and that RAN dipeptides and NMJ dysfunction are drivers of *C9orf72* disease pathogenesis. These AAV-mediated models of *C9orf72*-associated ALS/FTD will be useful tools for studying disease pathophysiology and developing new therapeutic approaches.

## Abstract (precis)

**Summary:** *C9orf72*-linked motor neuron disease models with viral-mediated expression of GGGGCC repeat expansion in mice show neuropathology and behavioural deficits.

## INTRODUCTION

Hexanucleotide repeat expansions (HREs) in *C9orf72* are regarded as the most common genetic cause of the progressive neurodegenerative diseases, amyotrophic lateral sclerosis (ALS) and frontotemporal dementia (FTD). ALS primarily affects motor neurons and leads to progressive failure of the neuromuscular system with muscle wasting and paralysis, while frontotemporal dementia (FTD) is caused by the degeneration of neurons in the frontal and temporal lobes, leading to cognitive deficits. GGGGCC (G_4_C_2_) repeat expansions in the first intron of *C9orf72* are present in up to 40% of familial ALS and 25% of familial FTD patients, and are also present in 5-20% of sporadic ALS patients (DeJesus-Hernandez et al., 2011; Renton et al., 2011, 2014; Majounie et al., 2012). Normal healthy *C9orf72* alleles contain between 2 and 30 repeats, whereas disease-associated alleles vary in size and can exceed several thousand HREs (DeJesus-Hernandez et al., 2011; Renton et al., 2011). Three different pathological alterations providing clues to the etiology of *C9orf72*-related disease can be found in post-mortem tissue from patients with HREs: first, a reduction in all three mRNA transcripts generated from *C9orf72*, suggesting that disease may arise by a loss-of-function mechanism (DeJesus-Hernandez et al., 2011; Belzil et al., 2013; Donnelly et al., 2013); second, formation of intracellular foci by aggregation of RNA expansions; and third, dipeptide repeat proteins (DPRs) which are generated from long expansions via repeat-associated non ATG-dependent (RAN) translation (Cooper-Knock et al., 2014; Schludi et al., 2015).

Recent studies suggest that G_4_C_2_ expansions and DPRs may induce cell toxicity by sequestration of RNA binding proteins (Cooper-Knock et al., 2014; Lee et al., 2016) and disruption of nucleocytoplasmic transport (Zhang et al., 2015; Freibaum et al., 2015). Models designed to mimic either *C9orf72* haploinsufficiency or toxic gain of function have been developed to identify the combination of factors inducing relevant disease pathology. While *C9orf72*-knockout mice display motor and cognitive deficits along with heightened neuroinflammation, these mice do not exhibit any significant neuronal pathology, suggesting that a simple loss-of-function mechanism is unlikely to be the sole cause of *C9orf72*-linked neurodegeneration (Koppers et al., 2015; O'Rourke et al., 2016). Four different toxic gain-of-function mouse models developed through expression of patient-derived *C9orf72* containing hundreds of HREs consistently displayed intracellular RNA foci and DPRs in the CNS, though they differed in the generation of ALS/FTD disease phenotypes (Jiang et al., 2016; Liu et al., 2016; O'Rourke et al., 2015; Peters et al., 2015; Chew et al., 2015). In contrast, acute AAV-mediated, actin-promoter-driven expression of 66 repeats in mice recapitulates ALS-like pathology and induces cognitive and behavioural deficits, despite the lack of any flanking *C9orf72* sequence (Chew et al., 2015).

Interruptions in repeat expansions are found in alleles for spinocerebellar ataxia (SCA) (Chung et al., 1993; Pearson et al., 1998a; Matsuura et al., 2006), myotonic dystrophy type 1 (Musova et al., 2009), Friedreich ataxia (Stolle et al., 2008) and fragile X syndromes (Eichler et al., 1994; Pearson et al., 1998a), although the consequences of the interruptions on disease etiology remain unclear. Interrupting the expression of pure glycine-alanine (GA) dipeptide repeats with proline residues in mice prevented the formation of dense ubiquitin-positive inclusions and the development of behavioural phenotypes and pathological hallmarks consistent with C9orf72-ALS/FTD (Zhang et al., 2016). An interruption in the expression of DPRs created by inserting stop codons between G_4_C_2_ repeats in *Drosophila* blocked DPR formation and neurodegeneration but preserved RNA foci generation demonstrating that DPRs are toxic (Mizielinska et al., 2014). Here, we generated mice expressing HREs, utilizing AAV serotype 9 (AAV9) to achieve an acute, widespread expression following cisterna magna delivery in postnatal day 1 (P1) pups. We compared mice expressing 10 HRE expansions with mice expressing 102 repeats interrupted at regular intervals to examine the effects of interrupted expansions on disease etiology. We show that both AAV-(G_4_C_2_)^10^ and AAV-(G_4_C_2_)^102^ repeats lead to the formation of a similar number of intranuclear RNA foci in the CNS *in vivo*, but only (G_4_C_2_)^102^ transgenic mice generate DPRs and p62 inclusions, have Purkinje cell apoptosis, exhibit neuromuscular junction pathology, and display gait and cognitive deficits.

## RESULTS

### Injection of AAV9 HREs repeats leads to intranuclear RNA foci *in vivo*

**Fig. 1.**
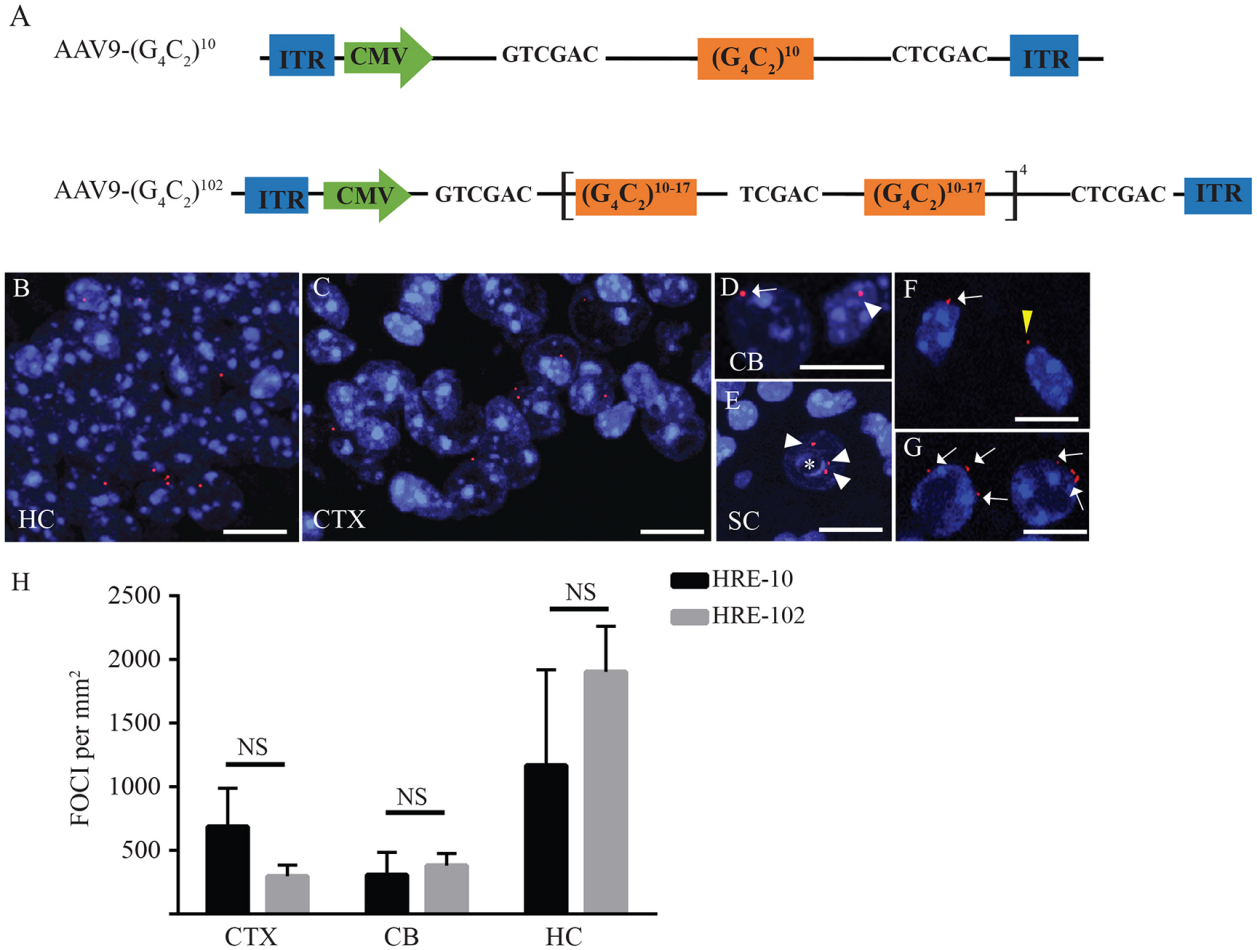
**AAV9 hexanucleotide RNA expansions** (**HREs) can generate RNA foci in the mouse CNS.** (A) Two different HREs were designed and subcloned into AAV9 vectors with the (G_4_C_2_)^102^ expansions constructed using a mixture of (G_4_C_2_)^10-17^ oligonucleotides with an intervening linker sequence. (B-G) RNA fluorescence *in situ* hybridization was used to detect RNA foci, which were abundant in the hippocampus (HC, B), cortex (CTX, C), cerebellum (CB, D) and sparse in the spinal cord (SC, E). Asterisk in E indicates a motor neuron. The majority of the foci were intranuclear (D,E, white arrowheads), although cytoplasmic (F, yellow arrowhead) and juxtanuclear foci (F,G, arrows) were detected. (H) There were no significant differences (NS) in the number of foci detected per mm^2^ in either the HC, CTX or CB between HRE-10 and HRE-102 mice as assessed using an unpaired two-tailed Student's *t*-test for each region (HRE-10, *n*=6; HRE-102, *n*=6). Scale bar: 10 µm. Nuclei counterstained in blue with Hoechst 33342.

In these studies, we used self-complementary AAV9, due to its enhanced CNS transduction efficiency compared with single-stranded AAV9 (McCarty et al., 2001) and its ability to achieve transduction in multiple regions of the mouse brain (Lukashchuk et al., 2016). To generate mice expressing HREs, we first constructed two separate AAV9 vectors containing either 10 or 102 HREs (Fig. 1A). The AAV9-(G_4_C_2_)^102^ (referred to as HRE-102) construct was made by ligating between 10-17 HREs separated by a short TCGAG linker sequence, which interrupts the sequence without introducing a stop codon, resulting in mixed dipeptide proteins. The advantages to using this approach are: firstly, the interruptions potentially stabilized the sequence, reducing the risk of DNA rearrangements between the flanking inverted terminal repeats (ITRs) of the AAV; secondly, we could test whether *C9orf72*-mediated pathological hallmarks, such as DPRs, p62 inclusions and TDP-43 aggregation, still persist in the presence of interrupted repeat sequences.

Mice expressing repeat expansions were generated by delivering AAV9-HREs to early postnatal (P1) mice via the cisterna magna, which resulted in widespread transduction of the adult mouse brain as described in our previous work (Lukashchuk et al., 2016). Twelve months after AAV9 delivery, mice were killed and different brain and spinal cord tissue analysed using RNA fluorescent *in situ* hybridization (FISH) to see whether intracellular RNA foci were present. We found that the regions with the highest number of RNA foci per area were the brain stem, Purkinje cell layer of the cerebellum, CA1 and hilus of the hippocampus, midbrain and cortex (Fig. 1B-D). RNA foci were also detected in the spinal cord, even though they were less numerous in this region (Fig. 1E). The majority of RNA foci were intranuclear (Fig. 1D-E, white arrowhead), but we also detected perinuclear foci (Fig. 1F,G, arrows) and occasionally observed cytoplasmic foci (Fig. 1F, yellow arrowhead). There were no significant differences in the number of RNA foci between the HRE-10 and HRE-102 mice in any of the regions examined (Fig. 1H).

### HRE-102 mice exhibit extensive DPR pathology, enhanced p62 expression and dispersed CA1 neurons

**Fig. 2.**
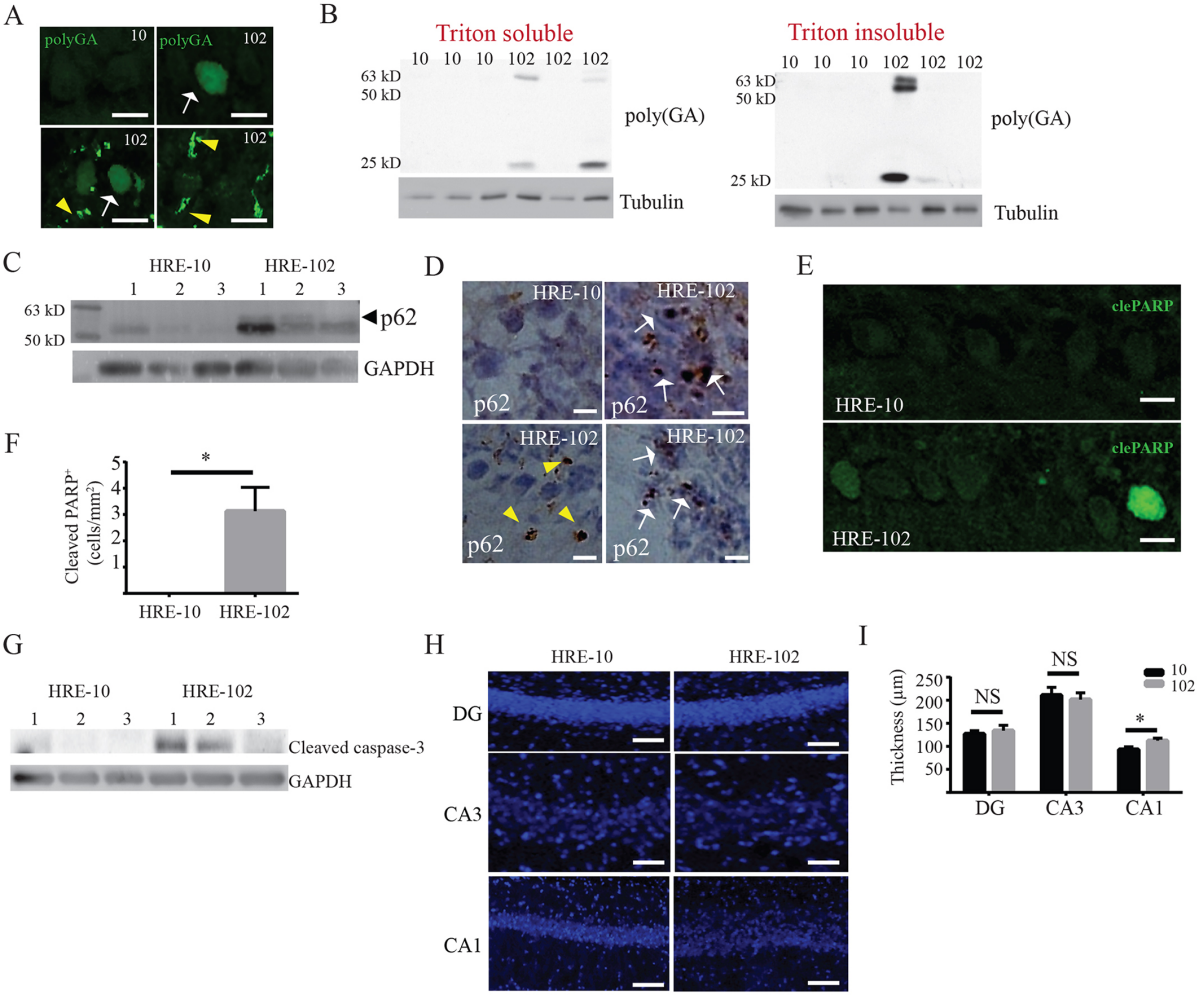
**Dipeptide protein repeat (DPR) pathology, hippocampal CA1 dispersal and upregulation of cell death markers are observed in HRE-102 mice.** (A) Cytoplasmic and nuclear DPRs adopt several morphologies in a subset of HRE-102 mice using a poly-GA antibody in cerebellar tissue. Arrows indicate nuclear expression and arrowheads show discrete cytoplasmic staining. (B) Immunoblots of cerebellar lysates show that both Triton-soluble and Triton-insoluble proteins contain GA-rich DPRs in HRE-102 mice that were never observed in HRE-10 mice. (C) HRE-102 mice show increased p62 expression in cerebellar tissue compared with HRE-10 mice; arrowhead shows 60 kDa isoform that was consistently absent in HRE-10 brains. (D) p62 inclusions are evident in both the nucleus (arrows) and cytoplasm (arrowheads) in the cerebellum of HRE-102 mice but are absent in HRE-10 mice. (E,F) A significant number of Purkinje cells in the cerebellum of HRE-102 mice show signs of early apoptosis reflected by cleaved PARP immunoreactivity (*n*=4 for both, *P*=0.046). (G) Enhanced cleaved caspase-3 expression is observed in cerebellar lysates from HRE-102 mice. (H,I) There were no differences in the number of neurons as determined by NeuN staining in the dentate gyrus (DG: HRE-10, *n*=4; HRE-102, *n*=6; *P*=0.66) or CA3 (HRE-10, *n*=4; HRE-102, *n*=6; *P*=0.69) in the hippocampus between HRE-10 and HRE-102, but there was a significant dispersal of CA1 neurons in HRE-102 mice (HRE-10, *n*=4; HRE-102, *n*=6; *P*=0.046). Nuclei in H are counterstained blue with Hoechst 33342. All comparisons were made with an unpaired two-tailed Student's *t*-test with Welch's correction; mean±s.e.m. Scale bars: 10 µm (A,E,H) and 25 µm (D).

To test whether the AAV9-engineered mouse model develops other pathological hallmarks of ALS/FTD such as *C9orf72*-derived RAN (c9RAN) protein aggregates, TDP-43 inclusions and neuronal loss, we used an anti-poly-GA antibody (Mackenzie et al., 2013) to probe for glycine-alanine-rich DPRs in mouse brains. As shown in Fig. 2, we detected either a homogenous nuclear expression (arrow) or an intense, discrete staining (arrowhead) within and outside the nucleus (Fig. 2A). These poly-GA pathologies were most widely distributed in the cerebellum and brain stem, and visible only in HRE-102-treated mice (poly-GA detected in 4 out of 6 mice), and absent in HRE-10-expressing mice (poly-GA detected in 0 out of 6 mice). Triton-insoluble and Triton-soluble cerebellar/brain stem lysates were found to contain several poly-GA peptides ranging from 25 to 63 kDa in a subset of HRE-102 mice, but not in HRE-10 mice (Fig. 2B). Additionally, the expression of p62, a marker of cargo destined for autophagic degradation (Bjorkoy et al., 2005; Komatsu et al., 2007) and c9RAN pathology (Mori et al., 2013a,b; Schludi et al., 2015), was enhanced in the cerebellum of HRE-102 mice (Fig. 2C), and was detected in dense intracellular (arrows) and extracellular (arrowheads) aggregates in HRE-102 mice (Fig. 2D).

Next, we examined the expression of cleaved poly (ADP-ribose) polymerase (PARP) and cleaved caspase-3 to see whether there was evidence of cells undergoing apoptosis. We focused our analysis on the cerebellum, due to the heightened DPR and p62 expression we observed in the HRE-102 mice (Fig. 2A-D), which has previously been found to cause cell toxicity (Lee et al., 2016; Lopez-Gonzalez et al., 2016; Tran et al., 2015). As shown in Fig. 2E-G, we found that cleaved PARP in cerebellar Purkinje cells (Fig. 2E-F) and cleaved caspase-3 expression (Fig. 2G) in the cerebellum were significantly increased in HRE-102 mice, indicating that these areas were in the early stages of cell death. Interestingly, despite the presence of RNA foci and c9RAN pathology, neither astrogliosis (Fig. S1) nor neurodegeneration in the cerebellum (Fig. S2C,D) was observed. This absence of repeat-length-dependent neurodegeneration was also observed in other regions such as the cortex (Fig. S2A-B), spinal cord (Fig. S2E-F) and hippocampus (Fig. 2H,I), although there was a significant dispersal in the CA1 (Fig. 2H,I) that is characteristic of *reeler* mutant mice (Howell et al., 1997; Trommsdorff et al., 1999). Additionally, although we were able to detect infrequent TDP-43 aggregates (Fig. S3A-B) in the cerebellum, cortex, midbrain and hippocampus of HRE-10 and HRE-102, there was no significant difference between mouse groups with respect to TDP-43 pathology. We also did not detect any differences between the groups with respect to phosphorylated TDP-43 pathology (J.S.C., unpublished observations). In contrast to the largely nuclear TDP-43 pathology reported by Chew et al. (2015), the majority of TDP-43 aggregates in both groups of AAV-HRE-injected mice were cytoplasmic (Fig. S3C).

### HRE-102 mice develop disease-related neuromuscular junction (NMJ) pathology

**Fig. 3.**
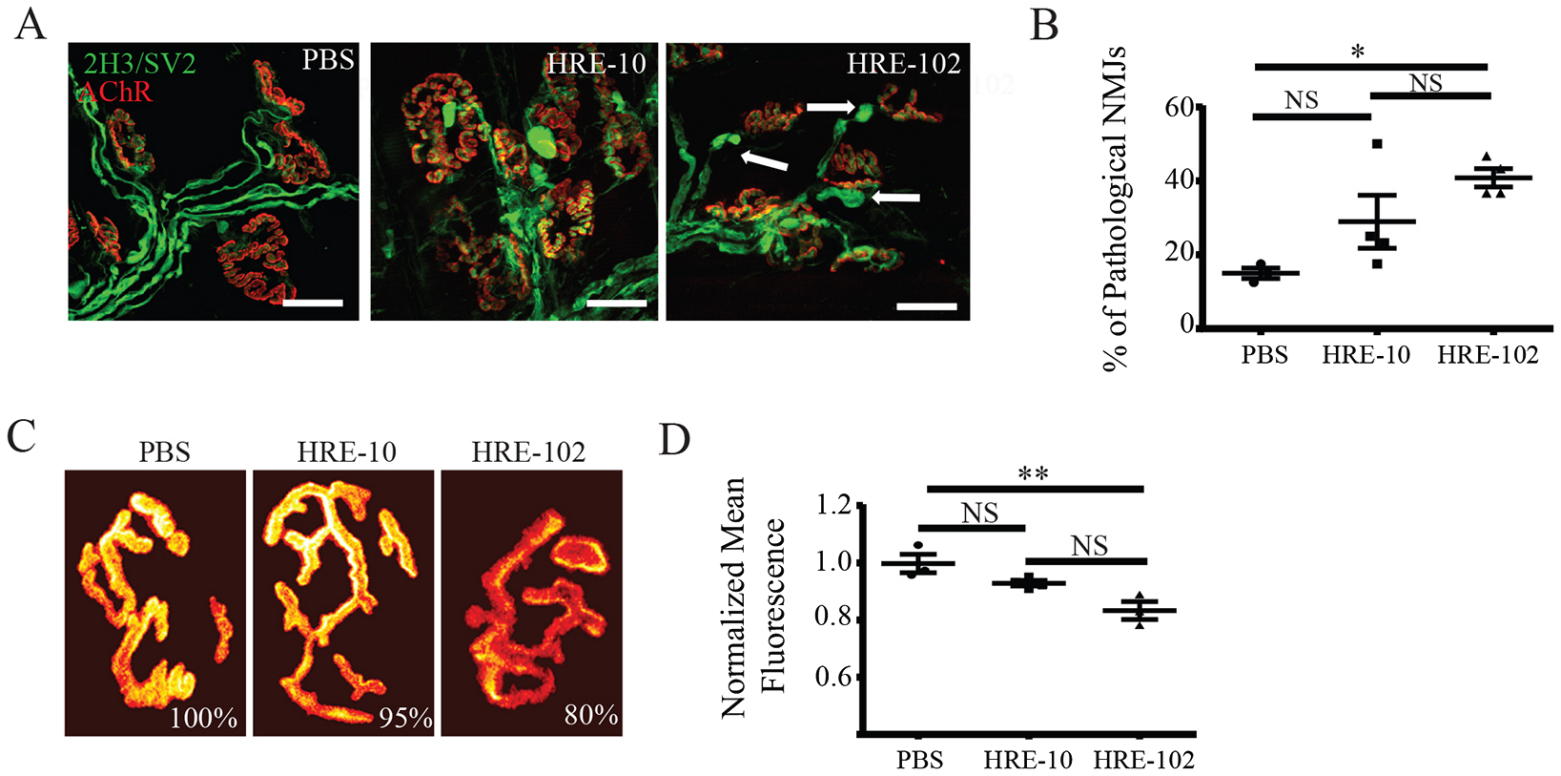
**Neuromuscular junction (NMJ) defects are present in the HRE-102 mice.** (A,B) Abnormal neurofilament accumulation and nerve terminal blebbings (arrows) are prominent in HRE-102 mice (PBS, *n*=3; HRE-10, *n*=4; HRE-102, *n*=4; where *n*=individual muscles per genotype; *F*_2,8_=6.47, *P=0.02, *post hoc* Tukey: HRE-10 vs PBS, *P*=0.19; HRE-10 vs HRE-102, *P*=0.23; HRE-102 vs PBS, *P*=0.02). Scale bar: 30 µm. (C,D) HRE-102 mice have a significant decrease in acetylcholine receptor density compared with PBS-injected controls (*F*_2,7_=10.79, **P=0.007, *post hoc* Tukey: HRE-10 vs PBS, *P*=0.16; HRE-10 vs HRE-102, *P*=0.056; HRE-102 vs PBS, *P*=0.006). The fluorescence intensity (total fluorescence of that synapse divided by the area of that given synapse) of a saturating dose of α-bungarotoxin Alexa Fluor 594 was measured and normalized to PBS controls. All comparisons were made with a one-way ANOVA and *post hoc* Tukey for comparisons between groups; mean±s.e.m. NS, not significant.

In the absence of a significant loss of neuronal cell bodies in the CNS in both mouse cohorts and since NMJ pathology is present in several different mouse models of ALS (Iguchi et al., 2013; Frey et al., 2000; Murray et al., 2010; Bennett et al., 2014), we asked whether peripheral neuromuscular junctions were altered in the HRE-102-expressing mice. To do this, muscles from 12-month-old mice were double-labelled with an anti-neurofilament antibody to label nerve terminals and fluorescent bungarotoxin to label acetylcholine receptors (AChRs), and NMJs were imaged with a confocal microscope. A close inspection of NMJs revealed a significant increase in pathological NMJs (abnormal pre-synaptic neurofilament accumulations and/or nerve terminal blebbings) in HRE-102 mice (Fig. 3A,B), indicating that the NMJ may be an early target of *C9orf72*-related toxicity. It is well established that the density of postsynaptic acetylcholine receptors (AChRs) is significantly reduced in mouse models of motor neurons disease and myasthenia gravis (Losen et al., 2005; Kong et al., 2009). Here, we asked whether the density of AChRs at NMJs of HRE-102 mice is also altered. AChRs were labelled with BTX-594 and the density of AChRs at NMJs of HRE-102, HRE-10 and PBS was compared (Fig. 3C,D). The total fluorescence of AChRs (fluorescence of a synapse per area of the synapse) was decreased by 20% in muscles expressing HRE-102 compared with NMJs of PBS-injected mice. These results indicate that HRE-102 expression reduces the density of AChRs at postsynaptic sites. Collectively, HRE-102 mice exhibit a loss in NMJ integrity at 12 months of age that is consistent with the early stages of motor neuron degeneration.

#### HRE-102 mice have progressive gait and behavioural deficits

**Fig. 4.**
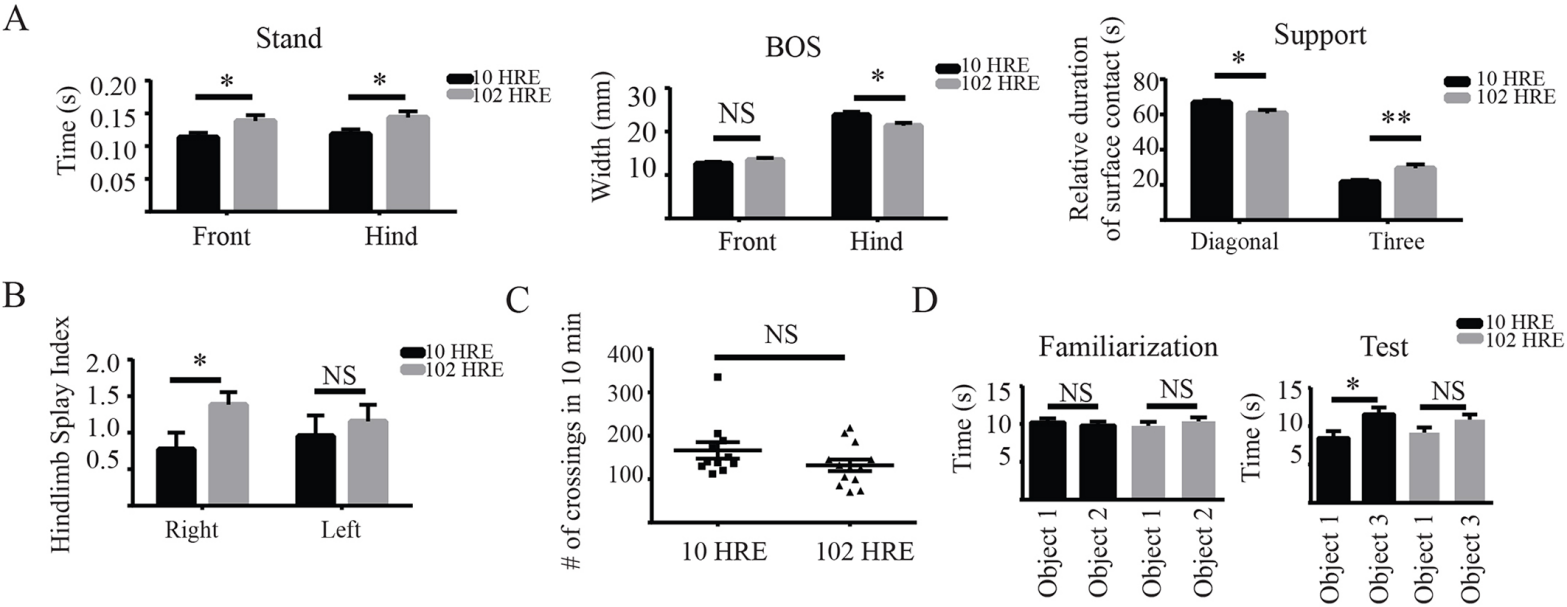
**Gait and cognitive deficits observed in HRE-102 mice are absent in HRE-10 mice in 12-month-old mice.** (A) Gait abnormalities such as the front and hind stand index, the hind base of support (BOS), the diagonal support and the three-legged support were observed using a Catwalk system in HRE-102 mice (HRE-10, *n*=11; HRE-102, *n*=13 for each group; front stand, *P=0.04; hind stand, *P=0.04; front BOS, *P*=0.19; hind BOS, *P=0.02; diagonal support, *P=0.01; three-legged support, **P=0.006). (B) HRE-102 mice (*n*=13) had a moderate splay in the right (*P=0.04) but not left (*P*=0.59) hind limb compared with HRE-10 mice (*n*=11). (C) There were no significant differences (NS) in activity (*P*=0.15) between HRE-10 (*n*=11) and HRE-102 (*n*=13) in the open field. (D) An object recognition test identified a cognitive deficit in HRE-102 mice as they failed to discriminate in the testing phase (*n*=13; familiarization phase, *P*=0.43; testing phase, *P*=0.09) between a novel object and an object previously explored in the familiarization phase unlike HRE-10 mice (*n*=11, familiarization phase, *P*=0.57; testing phase, *P=0.02). All comparisons were made with an unpaired two-tailed Student's *t*-test with Welch's correction; mean±s.e.m. NS, not significant.

To assess whether the c9RAN and NMJ pathology have any consequences on mouse behaviour, we analysed motor and cognitive tasks. We used a Catwalk system to examine different parameters measuring motor coordination and gait in ambulatory mice, and found that HRE-102 mice had gait abnormalities, with significantly irregular gait patterns compared with HRE-10 mice in the stand, base of support (BOS), diagonal support and three support parameters (Fig. 4A). When we assessed the hindlimb splay in the two cohorts (HRE-10 and HRE-102), we found that there was a significant defect in the right hindlimb in the HRE-102 mice (Fig. 4B). These deficits did not appear to affect the exploratory activity of the mice as HRE-102 mice were not significantly different to HRE-10 mice in the open field at 12 months of age (Fig. 4C). When we analysed data between 6 and 12 months of age from several of the same tasks, we noted that most of the gait parameters, including those measuring stride irregularities (stand, swing, and swing speed), in addition to activity in the open field showed significant deterioration with age in the HRE-102 mice (Fig. S4A-D), demonstrating that the behavioural impairments in HRE-102 mice were progressive. There were, however, no differences between groups in the accelerating rotarod over the 10 months of testing (Fig. S4E). We next used the object recognition paradigm to determine whether the HRE-102 mice had any cognitive deficits that could correlate with the CA1 dispersal demonstrated in Fig. 2D. This behavioural task exploits the innate interest of mice to explore a novel object by forcing mice to discriminate between a novel object and an object previously explored. Both sets of mice explored two novel objects without any significant difference in the familiarization phase (Fig. 4D), but only HRE-102 mice were unable to discriminate between a novel object and one they had previously explored, indicating the presence of a memory deficit.

## DISCUSSION

Current mouse models of *C9orf72*-related ALS/FTD have supported a toxic gain-of-function mechanism by which long G_4_C_2_ HREs induce the formation of intracellular RNA foci and RAN protein aggregates, leading to toxicity and behavioural dysfunction. Here, we used AAVs to express either 10 or 102 HREs containing regularly spaced interruptions in mice, and demonstrate that while both lines of mice have similar numbers of RNA foci dispersed through the brain, only HRE-102 mice generate c9RAN pathology, exhibit NMJ pathology, hippocampal CA1 dispersion, early markers of apoptosis, and display gait and cognitive deficits.

To date, four independent mouse models expressing either full-length or truncated human *C9orf72* with hundreds of HREs have been generated, along with an AAV-mediated model expressing a reduced number (66) of HREs. All of these mouse models have detectable RNA foci and c9RAN aggregates in the brain, although the minimum size necessary to induce RNA foci formation differs widely. Expression of a full-length *C9orf72* bacterial artificial chromosome (BAC) transgene in mice was unable to induce RNA foci formation with 36-37 HREs (Liu et al., 2016; O'Rourke et al., 2015), and in mice expressing a truncated *C9orf72*, expression of 100 G_4_C_2_ expansions yield limited RNA foci (Jiang et al., 2016). In contrast, AAV-mediated expression of 66 HREs with a constitutive actin promoter generated significant ALS/FTD pathology, which was absent when only 2 HREs were expressed (Chew et al., 2015). We chose a higher minimum expansion length of 10 HREs to determine whether we could cross the minimal threshold for generating RNA foci without forming DPRs. Surprisingly, our mice expressing HRE-10 showed similar numbers of RNA foci in the CNS (Fig. 1H) to our mice expressing HRE-102, despite the presence of DPRs in only the HRE-102 mice. We therefore conclude that RNA foci density is not an accurate predictor of DPR formation.

The presence of poly-GA and p62 aggregates (Fig. 2A-D) in our HRE-102 mice indicates that the interruptions we introduced between every 10-17 GGGGCC repeats do not prevent DPR formation. In contrast, interrupting AAV-mediated poly-GA expression in mice with proline residues (Zhang et al., 2016) or inserting stop codons between extended HREs expressed in *Drosophila melanogaster* (Mizielinska et al., 2014) abolish DPR formation and prevent other disease-related pathologies and behavioural phenotypes. Our interrupted HRE-102 construct shifted the reading frame of the translated sequence without introducing a stop codon, resulting in the formation of mixed dipeptides as opposed to single extended tracts of poly-GA, poly-GR or poly-GP. We were unable to detect robust poly-GR or poly-GP expression in our mice, although this is more likely to be due to the cross-reactivity of our antibody to endogenous proteins in mouse tissue, as a cell culture model expressing an inducible version of our HRE-102 construct detected poly-GA, poly-GP and poly-GR DPRs (Stopford et al., 2017).

Interruptions in *C9orf72* expansions have not yet been described, but interrupted repeat expansions have frequently been detected in other neurological disorders, including spinocerebellar ataxia (Chung et al., 1993), fragile X syndrome (Eichler et al., 1994), myotonic dystrophy type I (Musova et al., 2009) and Friedreich ataxia (Stolle et al., 2008). There is no consensus on the general effects of the interruptions on disease progression, although there is strong evidence that interruptions can stabilize DNA sequences and prevent the formation of slipped DNA structures in CAG and CGG repeat disorders (Eichler et al., 1994; Pearson et al., 1998a,b; Menon et al., 2013). The more rapid development of ALS/FTD phenotypes in the uninterrupted HRE-66 mice described by Chew et al. (2015) compared with our interrupted HRE-102 may reflect a stabilizing presence of our interruptions on the expansion sequence.

In large GAA expansions in Friedreich ataxia, interruptions can stabilize the DNA sequence (Pollard et al., 2004) without necessarily having any effect on the age of onset (Stolle et al., 2008). In contrast, interruptions in the CTG expansions in myotonic dystrophy type I delay disease onset and suppress the muscular dystrophic phenotype (Musova et al., 2009), disruptions in the CGG repeats of fragile X mental retardation 1 (*FMR1*) protect carriers from premature ovarian failure (Bodega et al., 2006), and CAT insertions between CAG expansions in the ataxin-1 gene (*ATXN1*) prevent the development of the full SCA1 phenotype. There is also evidence that interruptions can lead to the presentation of new phenotypes as disruptions in the ATTCT repeats in *SCA10* are associated with a higher incidence of seizures (Matsuura et al., 2006) and CAG repeats that normally cause SCA2 can lead to parkinsonism when CAA triplets interrupt the CAG expansion (Charles et al., 2007). As a result, it is difficult to draw conclusions on the effects of the interruptions on disease progression in our AAV HRE-102 mice, although it is notable that these mice develop some of the pathologies and behavioural phenotypes consistent with ALS/FTD, suggesting it is less likely that the interruptions led to a novel phenotype. It also seems unlikely that the interruptions in our HRE-102 mice disrupt protein aggregation as in the mice described by Zhang et al. (2016), which harbour proline interruptions between DPRs, as we detected clear poly-GA and p62 depositions in the tissue (Fig. 2A-D).

We did not observe extensive TDP-43 pathology in either the HRE-10 or HRE-102 mice, and the majority of TDP-43 depositions were cytoplasmic (Fig. S3), which contrasts with the nuclear TDP-43 pathology in the HRE-66 mice described by Chew et al. (2015). The relative paucity of TDP-43 aggregates in the nucleus of our HRE-102 mice may be due to the interruptions disrupting the G-quadruplexes formed by pure G_4_C_2_ repeats and preventing the reported association between the G-quadruplexes and mRNA export proteins SRSF1 (Reddy et al., 2013; Zamiri et al., 2014) and hnRNPs (Conlon et al., 2016; Haeusler et al., 2014; Zamiri et al., 2014). We can also not rule out the possibility that an uninterrupted RNA sequence influences TDP-43 deposition as a result of increased toxicity. Future investigations on the effects of interruptions on G-quadruplexes and TDP-43 export will be necessary to clarify this.

As progressive deficits in spatial learning and memory and have been reported in *C9orf72* BAC transgenic mice expressing 450 repeats (Jiang et al., 2016), we carefully examined hippocampal pathology in the AAV-mediated mouse models since our HRE-102 mice failed to discriminate between familiar and novel objects, demonstrating the presence of a cognitive deficit (Fig. 4D). Previously, DPRs in the hippocampal CA1 of patients with *C9orf72* repeats have been reported (Pletnikova et al., 2014), but while we were unable to detect DPR aggregates in the hippocampus, we did observe a dispersal of the CA1 (Fig. 2H,I) in the HRE-102 mice that was reminiscent of the aberrant neuronal migration phenotype of *reeler* (Disabled-1) mouse mutants that occurs during early development (Trommsdorff et al., 1999; Howell et al., 1997). Interestingly knockdown of Reelin, the glycoprotein absent in *reeler* mutant mice, impairs rats in the object recognition task, whereas Reelin overexpression can rescue deficits in the object recognition task in mouse models of Alzheimer's disease (Brosda et al., 2011; Pujadas et al., 2014). Further analysis will be necessary to determine whether any link between Reelin and *C9orf72* exists.

In summary, these data support a HRE-length-dependent gain of toxicity, with c9RAN formation being the likely driver of pathogenesis because the gait, motor and cognitive deficits precede any major TDP-43 pathology. AAV-mediated expression of 102 GGGGCC repeats in mice leads to significant disease-related pathology despite the presence of interruptions leading to a mixture of translated DPRs. Our model provides an interesting comparison to similar AAV-mediated models expressing pure repeat expansions, and may help uncouple the relationship between DPR formation and disease pathophysiology.

## MATERIALS AND METHODS

### Antibodies and reagents

Rabbit anti-AAV VP1, VP2, VP3 (1:800, American Research Products, 03-61084), rabbit anti-NeuN (1:500, Cell Signaling, clone D3S3I), rabbit anti-GFAP (1:2000, Dako, Z0334), rabbit anti-TDP-43 (1:500, Proteintech, 18-280-1-AP), mouse anti-TDP43-phospho Ser409/410-1 (1:4000, Cosmo Bio, CAC-TIP-PTD-M01), mouse anti-Calbindin (1:1000, Sigma, C9848), mouse anti-p62 lck ligand (1:1000 for WB, 1:250 for ICC, BD Biosciences, clone 3, 610833), rabbit anti-α-tubulin (1:5000, Abcam, 4074), rabbit-cleaved-caspase 3 (1:1000, Cell Signaling, 9661), mouse anti-cleaved PARP (1:250, Cell Signaling, 9548), mouse anti-GAPDH (1:5000, Calbiochem, CB 1001), mouse anti-poly(GA) [1:500 for WB, 1:5000 for ICC, clone 5F2 (Mackenzie et al., 2013) kindly provided by Dieter Edbauer] were used at the dilutions listed.

### Cell lines and culture

HEK 293T cells were maintained in growth medium (GM) consisting of Dulbecco's modified Eagle's medium (DMEM; Sigma, D5796) supplemented with 10% fetal bovine serum (Sigma, USA), penicillin (100 U/ml), and streptomycin (100 U/ml) at 37°C and 5% CO_2_.

### Generation of repeat expansion constructs

Sense [TCGAC (G_4_C_2_)^10^] and antisense [ACGT(G_4_C_2_)^10^] oligonucleotides (Sigma) with *Sal*I/*Xho*I overhangs were annealed by denaturing at 95°C for 5 min and cooling at 0.5°C/min to 25°C. The dsDNA (G_4_C_2_)^10^ was ligated into *Sal*I- and *Xho*I-digested pcDNA6.2-GW/EmGFP-miR (Invitrogen) to generate pcDNA6.2-GW-(G_4_C_2_)^10^. Further (G_4_C_2_)*^n^* repeats where *n* varied between 10 and 17 repeats were subcloned into the *Xho*I site to generate the longer pcDNA6.2-(G_4_C_2_)^102^ construct. The (G_4_C_2_)^10^ and (G_4_C_2_)^102^ constructs were subsequently sub-cloned into a pAV2-CMV (cytomegalovirus) plasmid and authenticity was confirmed by sequencing. Transformations of plasmids containing the (G_4_C_2_)*^n^* repeat constructs were performed using recombination-deficient β-10 *E. coli* (NEB) to minimize any rearrangements.

### Large-scale scAAV9 production

Sixty 15 cm plates containing HEK293 cells at 80% confluence were transfected using polyethylenimine (MW∼25 K) with a mixture of three plasmids required to generate an infectious scAAV9 viral particle: (1) a plasmid providing helper genes isolated from adenovirus that enhance viral infectivity (pHelper); (2) an ITR-containing a plasmid carrying the gene of interest [pAV2-CMV-(G4C2)*^n^*; we used the pAV2-CMV-GFP consisting of two ITRs in a truncated genome that resulted in a self-complementary AAV9 (scAAV9), as described by others (McCarty et al., 2001)]; (3) a plasmid that carries the AAV Rep-Cap proteins (pAAV2/9). Each 15 cm plate was transfected with 40 µg DNA comprising a 2:1:1 molar ratio of pHelper:pAV2-CMV-(G_4_C_2_)*^n^*:pAAV2/9. Four days after transfection, the AAV-enriched medium was collected, incubated at 37°C for 2 h with 3750 units of benzonase nuclease (Sigma), filtered through a 0.22 µm filter, and concentrated to a volume of 1 ml using Amicon spin filter units (Millipore). The virus was then washed with 50 ml phosphate-buffered saline (PBS, pH 7.3) in the same Amicon spin filter units, and concentrated to a final volume of 0.5 ml. The viral sample volume was expanded to 14 ml with PBS and separated through a discontinuous iodixanol (Sigma, D1556) gradient (4 ml of 54%, 9 ml of 40%, 9 ml of 25%, 5 ml of 15%), and centrifuged at 69,000 rpm for 1.5 h at 18°C. Phenol Red was added to the 54% and 25% iodixanol layers to help with identifying the virus-enriched layer. The purified virus was found as a white layer between the 54% and 40% iodixanol gradient and subsequently removed in 0.5 ml fractions using a syringe, with 10 µl of each fraction mixed at an equal ratio with 2× reducing sample SDS-PAGE buffer, heated to 75°C for 20 min, separated on a 4-20% precast TGX mini-gel (Bio-Rad), and stained with Sypro-Ruby according to the manufacturer's protocol (Life Technologies). Fractions that showed a pure virus composed solely of the VP1, VP2 and VP3 bands were combined, and washed against five full volumes (15 ml each) of PBS with an Amicon spin filter, before collecting in a final volume of between 300 and 500 µl.

### Quantitative PCR to measure viral titres

Viral titres were determined with the Quantifast SyBR Green PCR Kit (Qiagen, 204054) on a Bio-Rad CFX96 thermal cycler, following the manufacturer's instructions. The number of copies in three dilutions of a purified AAV9 virus (1:100, 1:1000, 1:10,000) were compared with a standard curve generated by serial dilutions of a linearized pAV2-CMV-(G_4_C_2_)*^n^* vector. Primers used to quantify viral genomes were (PolyA, Forward: 5′-ATT TTA TGT TTC AGG TTC AGG GGG AGG TG-3′), (PolyA, Reverse: 5′-GCG CAG AGA GGG AGT GGA CTA GT-3′).

### RNA fluorescence *in situ* hybridization (FISH)

RNA FISH was performed as described previously (Cooper-Knock et al., 2014) with a few modifications. Briefly, a 5′ TYE-563-labelled LNA (16-mer fluorescent)-incorporated DNA probe was used against the sense RNA hexanucleotide repeat (Exiqon, batch number 607323). Coronal brain sections (20 µm) directly cut onto slides were air dried, and then incubated in ice-cold acetone for 10 min before washing in PBS. Slides were blocked with a hybridization solution [50% formamide, 2× saline sodium citrate (SSC), 100 mg/ml dextran sulphate, 50 mM sodium phosphate, pH 7.0] for 1 h at 66°C and then incubated with 400 ng/ml of denatured probe in hybridization solution overnight at 66°C. After hybridization, slides were washed once in 2×SSC/0.1% Tween-20 at room temperature and three times in 0.1× SSC at 65°C and counterstained with Hoechst 33342. All solutions were made with DEPC-treated water.

### Generation of the AAV9-mediated mouse model

Mice were kept in a 12 h dark:12 h light cycle, with free access to food and water and a standardized room temperature, based on the UK Home Office Code of practice for the housing and care of animals used in scientific Act 1986. All *in vivo* experiments carried out in this study were performed according to the Animal (Scientific Procedures) Act 1986, under Project License 40/3739, and were approved by the University of Sheffield Ethical Review Sub-committee and the UK Animal Procedures Committee (London, UK).

Viruses [4.5×10^10^ vg; scAAV9-CMV-(G_4_C_2_)^10^ or scAAV9-CMV- (G_4_C_2_)^102^] were injected into the cerebrospinal fluid (CSF) of postnatal 1 (P1) C57BL/6J wild-type mice, via the cisterna magna, under general anaesthesia. Pups from the same breeding pair were allocated randomly to the experimental groups. Viral delivery was carried out following the protocol described previously (Lukashchuk et al., 2016). Briefly, pups were anaesthetized in an induction chamber using 5% isoflurane and oxygen at 3 l/min before placing the animal over a red transilluminator in the prone position. Viruses were loaded in a Hamilton syringe attached to a peristaltic pump and injected into the cisterna magna, using a stereotaxic apparatus, at a flow rate of 1 µl/min to a maximum volume of 5 µl. Anaesthesia was maintained with 2% isoflurane and oxygen at 0.3 l/min during the injection.

### Immunohistochemistry

Twelve months after injection, animals were euthanized under terminal anaesthesia and transcardially perfused using a solution of PBS-heparin followed by 4% paraformaldehyde (PFA) in PBS. Brain and spinal cords were harvested and further post-fixed overnight in 4% PFA. After fixation, tissue was washed with PBS, cryoprotected in 30% of sucrose at 4°C, embedded in OCT (Cell Path) and snap frozen in liquid nitrogen. Brains and spinal cords were cut in 20-μm-thick coronal sections on a cryostat (Leica), placed directly onto silane-treated slides, and then air dried. Sections were incubated with gentle agitation for 2 h at room temperature in blocking buffer (PBS with 5% normal goat serum, 3% BSA and 0.2% Triton X-100) before incubating overnight at 4°C with the appropriate antibody diluted in PBS with 3% BSA. Following three washes with PBS, sections were incubated with an appropriate Alexa fluorophore-conjugated secondary antibody (1:1000; Invitrogen) for 1 h at room temperature, counterstained with Hoechst 33342 and mounted onto slides with Fluoromount (Sigma). For visualizing DPR pathology, slides were immersed in sodium citrate buffer (10 mM sodium citrate, 0.05% Tween 20, pH 6.0) for 30 min at 60°C before the blocking step. We used a Nissl stain (0.1% Cresyl Fast Violet; BDH, Lutterworth, UK) on spinal cord sections for counting motor neurons, and in cerebellar brain sections for visualizing p62 expression.

### Neuromuscular junction (NMJ) pathology

NMJ pathology was assessed on whole-mount immunohistochemically labelled preparations of hind paw lumbrical muscles from 1-year-old mice, as described previously (Powis et al., 2016; Wishart et al., 2014). Images were captured using a Nikon A1R confocal system combined with a Ti:E inverted microscope (×60 objective). Individual NMJs were assessed and abnormalities quantified by eye for evidence of neuromuscular pathology (presence of axonal blebbing/thinning or abnormal neurofilament accumulations in distal motor axons and/or motor nerve terminals). NMJ analysis was performed by investigators blinded to the treatment group.

### Quantitative fluorescence imaging

To assess the effect of CG repeats on the postsynaptic receptor density (marker of synapses), muscle cells were labelled with a saturating dose of fluorescent bungarotoxin (BTX) (5 μg/ml, ∼1 h) and images of NMJs were collected using IPLAB software as described previously (Akaaboune et al., 1999; Martinez-Pena et al., 2015). The fluorescence intensity of labelled AChRs was analysed with algorithms for MATLAB (MathWorks). Background fluorescence was determined by manual selection of a boundary region around each NMJ and subtracting it from the original image, and the mean of the total fluorescence intensity (which corresponds to total fluorescence of that synapse divided by the area of that given synapse) was measured (Turney et al., 1996; Akaaboune et al., 1999; Martinez-Pena et al., 2015).

### Immunoblotting

Adult mice were euthanized using an overdose of sodium pentobarbitol, and brains were rapidly dissected and snap frozen with liquid nitrogen. Brains were then homogenized in RIPA buffer using a glass Dounce homogenizer [150 mM NaCl, 50 mM Tris-HCl (pH 8), 0.5% sodium deoxycholate, 0.1% sodium dodecyl sulphate (SDS), 1% NP-40 supplemented with 1X EDTA-free protease inhibitor cocktail (Roche)] for 30 min on ice and centrifuged at 17,000 ***g*** at 4°C for 10 min. Supernatant was then collected as a cytoplasmic fraction and used to examine p62 reactivity. To examine poly-GA DPRs, the membrane-enriched pellet was further solubilized in a buffer containing 1% Triton X-100 and 0.5 M NaCl in 10 mM Tris-HCl (pH 7.4) for 45 min at 4°C with constant agitation and centrifuged at 17,000 ***g*** at 4°C for 10 min. Supernatant was collected as the triton-soluble fraction and the pellet was re-suspended in a buffer containing 2% SDS in 10 mM Tris-HCl (pH 7.4) and stored as the Triton-insoluble fraction. Protein lysates were mixed with a 4× Laemmli buffer with 5% β-mercaptoethanol, heated to 95°C for 5 min, separated by 10% SDS-PAGE and transferred onto a PVDF membrane (Millipore). Membranes were blocked with either 5% BSA or 5% non-fat dry milk in TBS with 0.05% Tween (TBST) for 30 min, before incubating at 4°C overnight with agitation with the appropriate antibody diluted in 5% BSA in TBST. Following three washes in TBST for 10 min each, membranes were incubated at room temperature with mild agitation with an appropriate secondary HRP antibody (Sigma, 1:5000) diluted in 5% BSA in TBST, washed three times with TBST for 10 min each, and developed with enhanced chemiluminescent substrate (Amersham-Pharmacia Biotech).

### Mouse behavioural tests

All mouse behavioural experiments were performed with the experimenter blind to the mouse cohort groups.

#### Open field

The open field was a 60 cm×40 cm×25 cm semi-transparent plastic box with a grid of 3×5 squares drawn on the underside of the box with black paint. Open field activity was measured in a room with minimal light for 10 min with the number of squares crossed recorded. A square crossing was defined as the passage of all four mouse paws over one of the boundaries of a square.

#### Object recognition

Mice were tested for their ability to remember novel objects using a paradigm described by others (Leger et al., 2013). Briefly, on day 1, mice were habituated in a rectangular 60 cm×40 cm×25 cm open field for 3 min. On the second day, mice were placed in the same box with two identical objects, and allowed to explore until a total of 20 s of exploration between the two objects had elapsed. Exploration was defined as mice actually sniffing, biting, touching with paws or examining the object. On the third day, one of the objects was replaced with a novel object, and the time measured at each object was recorded until a total of 20 s had elapsed. To make sure that the objects were interesting enough to entice mice to explore them, the first object was a standard red capped 75 cm^2^ tissue culture flask (Sigma, C7231) filled with a layer of purple dye treated agarose, while the second object was a stack of red slide boxes taped together.

#### Gait analysis

To analyse the gait pattern, 6- and 12-month-old mice were placed on the Catwalk XT (Noldus, The Netherlands), where they had to traverse a narrow chamber in the dark. Their footprints were automatically recorded with Catwalk 7.1 software from Noldus. Six runs were performed per animal and the three best (chosen for mice walking along the chamber path as opposed to repeatedly stopping) were selected for analysis. All results are expressed as the mean of three runs±s.e.m. The following parameters were measured and defined: (1) Stand: the time the paws spends in contact with the Catwalk surface. (2) Base of support (BOS): distance between either the front or hind paws. (3) Diagonal support: time spent on the Catwalk surface by a front paw and its opposing hind paw simultaneously (right front paw–left hind paw). (4) Three support: time spent on the Catwalk surface by three paws at the same time to support animal. (5) Swing: time between two consecutive placements in which the paw is not in contact with the Catwalk surface. (6) Swing speed: this parameter is calculated by dividing the stride length by the swing phase duration.

#### Hind limb splay

Twelve-month-old mice were suspended by the tail and the splay defects were observed and scored for right and left hind limbs by using the following scale: 0: normal splay; 1: mild defect; 2: moderate defect; 3: strong defect; 4: paralysed, as previously described (Mead et al., 2011). The results show the mean of each group±s.e.m.

#### Rotarod

Performance on the rotarod (Ugo Basile, 7650) was assessed every 2 weeks, twice per day, beginning 2 months after injection. Mice were initially trained for 3 consecutive days before the first trial. The rotarod was set to accelerate from 3 to 37 rpm in 300 s and the maximum time spent on the rotarod in a single trial out of two trials tested was recorded.

### Statistical analysis

Results are shown as mean±s.e.m. Student's unpaired *t*-test with a Welch's correction for two group comparisons was used to assess differences between HRE-10 and HRE-102 mice. For comparisons between three groups (PBS injected, HRE-10 and HRE-102 mice), a one-way analysis of variance (ANOVA) was used with Tukey's multiple comparison *post hoc* test when *P*<0.05. For assessing the effects of age on the gait and activity in each mouse cohort, we used a two-way ANOVA with a Šidák's test for multiple comparisons when *P*<0.05. For all tests, a *P* <0.05 was taken as statistically significant, and GraphPad Prism 6 was used for all statistical analyses.
